# Effects of 12-week high-intensity functional training on physical fitness in Wushu athletes

**DOI:** 10.1016/j.isci.2026.114985

**Published:** 2026-02-10

**Authors:** Xinzhi Wang, Kim Geok Soh, Shuzhen Ma, Fan Xu, Dong Zhang, Qian Lei

**Affiliations:** 1 Faculty of Sports Studies, Shandong University of Technology, Zibo 255000, Shandong, China; 2 Department of Sports Studies, Faculty of Educational Studies, University Putra Malaysia, Serdang 43400, Selangor, Malaysia; 3 School of Public Administration, Guilin University of Technology, Guilin 541004, China; 4 School of Physical Education, Yunnan Minzu University, Kunming 650500, China; 5 School of Physical Education, Quanzhou Normal University, Quanzhou, Fujian, China; 6 Faculty of Sports Studies, Aba Teachers University, Sichuan 623002, China

**Keywords:** Health sciences

## Abstract

High-intensity functional training (HIFT) has gained prominence in athletic preparation; however, its specific efficacy in male Wushu athletes remains undetermined. In this randomized controlled trial, sixty athletes were randomly assigned to an experimental group (EG) or a control group (CG). The EG undertook a structured 12-week HIFT intervention, comprising three 60-min sessions per week. The CG performed matched standard conditioning of equal duration and frequency, targeting 60%–70% HRmax. Physical fitness outcomes were assessed at baseline, week 6, and week 12, and data were analyzed using generalized estimating equations, with effect sizes expressed as model-based Cohen’s d. Compared to standard training, HIFT elicited significantly greater improvements in push-ups, standing long jump, rope jumping, and 30 m sprint performance (all *p* < 0.05, d = 0.57–0.93). The results demonstrate that HIFT can effectively enhance key performance-related fitness components in Wushu athletes, suggesting its utility as a conditioning strategy for combat sports.

## Highlights

• The study comparing control and experimental groups over a 12-week training period
• Key performance indicators were assessed to evaluate HIFT’s effectiveness
• Findings reveal significant improvements in endurance and strength

## Graphical abstract

**Figure:**
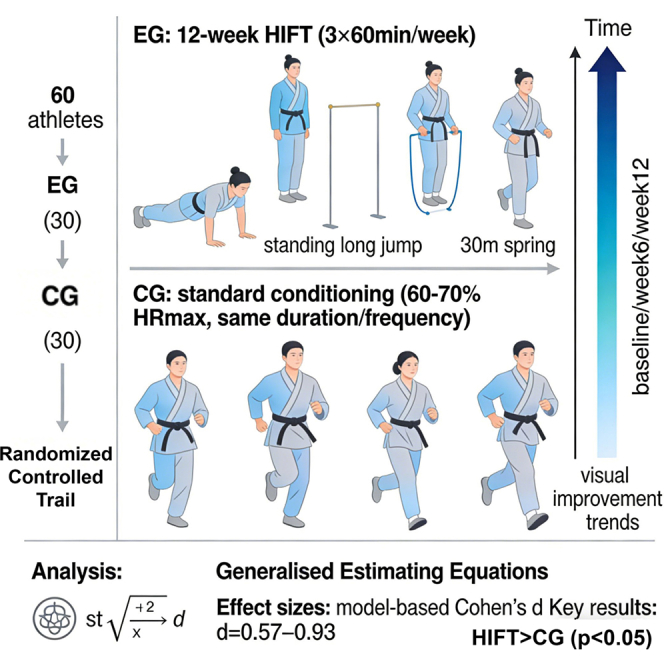

## Introduction

Wushu routines are highly demanding performance events that require athletes to combine explosive jumping techniques, rotational control, and aesthetic execution under conditions of fatigue. Common technical errors, such as insufficient jump height, incomplete rotations, and unstable landings, can substantially reduce competition scores. These deficiencies reflect underlying physical fitness constraints, particularly muscular endurance, lower-limb explosive performance, speed, flexibility, and neuromuscular coordination.^1^ Developing training methods that simultaneously target these capacities is therefore critical to advancing Wushu performance.^2^^,^^3^

High-intensity functional training (HIFT) has emerged as a multimodal conditioning method that integrates resistance exercises, plyometric drills, and metabolic intervals performed at relatively high intensity with minimal rest.^4^ Unlike traditional circuit training, which commonly follows fixed sequences of low- to moderate -ntensity exercises,^5^^,^^6^ HIFT emphasizes large multi-joint movements, functional movement patterns resembling sport skills, variable and often self-paced work volumes, and intensity prescription based on internal load markers such as heart rate or rating of perceived exertion.^7^ Additionally, HIFT differs conceptually from high-intensity interval training (HIIT), which primarily targets aerobic and anaerobic energy systems through cyclical activities such as running or cycling.^6^ By combining weightlifting derivatives, plyometrics, and calisthenic exercises, HIFT stimulates both aerobic and anaerobic systems, promotes neuromuscular coordination, and mirrors the complex, whole-body demands of combat sports.^7^ Recent evidence suggests that HIFT enhances muscle endurance, sprint performance, and explosive jump capacity in athletes from wrestling, Taekwondo, and volleyball.^8^^,^^9^^,^^10^ However, its role in Wushu training remains unexplored. HIFT’s emphasis on full-body functional resistance work theoretically makes it a suitable adjunct for building capacities relevant to Wushu physical fitness.

Importantly, the deficits observed in Wushu jumping techniques can be logically mapped onto HIFT elements.^11^ For instance, Olympic-lift derivatives and weighted plyometrics can increase vertical impulse, potentially correcting low jump height; gymnastic isometric holds and core exercises enhance trunk stability, improving landing control^12^^,^^13^; and short-duration, high-intensity circuits develop fatigue resistance, enabling athletes to sustain technical execution over prolonged routines.^14^ Such alignment suggests that HIFT may provide a targeted solution for performance-limiting weaknesses in Wushu.^15^^,^^16^ Previous research has shown that HIFT is widely adopted across various sports, such as Taekwondo,^9^ sambo,^17^ judo,^18^ soccer,^19^ wrestling,^20^ boxing,^21^ gymnastics,^22^ and volleyball.^23^ However, empirical studies evaluating HIFT in Wushu are scarce, and most existing research addresses isolated physical qualities rather than integrated performance outcomes. Moreover, no prior randomized trial has examined whether HIFT influences the technical execution of sport-specific jumping techniques.

To address this gap, the present study investigated the effects of a 12-week HIFT intervention on physical fitness outcomes in male Wushu routine athletes. We hypothesized that athletes performing HIFT would show greater improvements across (1) muscular endurance and horizontal jump performance, (2) coordination-related endurance, (3) sprinting speed, and (4) flexibility performance compared with athletes following standard conditioning. By linking Wushu-specific performance deficits to the functional and metabolic demands targeted by HIFT, this study aimed to provide sport-relevant evidence for integrating HIFT into technical preparation.

## Results

### Baseline comparisons and time-based changes in physical fitness variables

**Figure 1:**
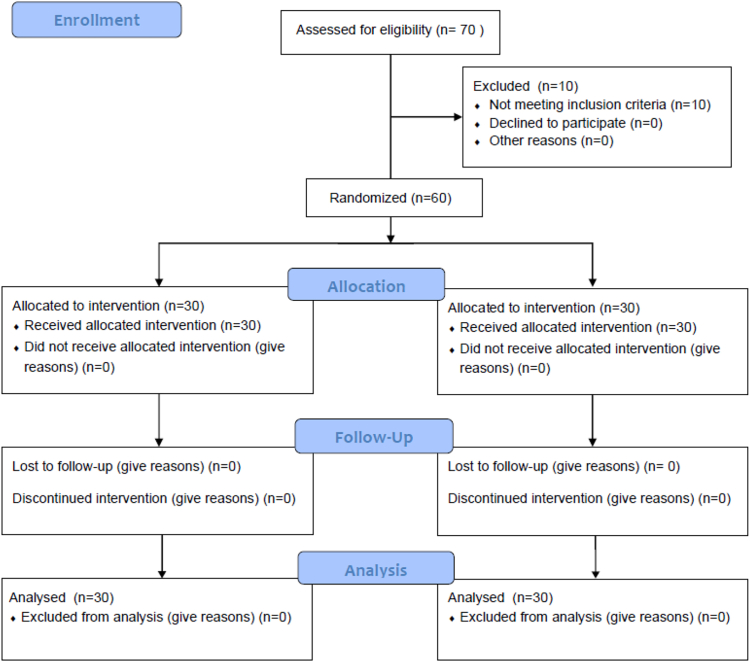
CONSORT diagram

The flow of participants through the study is presented in Figure 1. All randomized participants completed the intervention and were included in the final analysis. The experimental group underwent a structured 12-week HIFT program in addition to regular Wushu training, whereas the control group maintained their usual training routine. Table 1 summarizes the detailed training plan, including frequency, intensity progression, and exercise components.

**Table 1:** Information on training intervention

Experimental group	Intensity	Sets/reps	Rest (sets/reps)	Control group	Intensity	Sets/reps	Rest (sets/reps)
**Warm up (10 min)**							

Move the wrist, shoulder, hip, knee, and ankle	<60% HRmax	2 sets/4 reps	<10 s	move the wrist, shoulder, hip, knee, and ankle	<60% HRmax	2 sets/4 reps	<10 s

**Physical training (40 min)**							

Jumping rope Clean and jerk Jumping whirlwind kick Romanian deadlifts Jumping outward leg swing in flight Single-leg squat	90% HRmax	AMRAP	90 s/10 s	bench press deadlifts barbell biceps curl barbell squat sitting position with weight-bearing knee extension calf raises	60%–70% HRmax	3 sets/8–10 reps	60 s

**Cool down (10 min)**							

Muscle stretching (upper trapezius stretch, lunge with spinal twist, cobra pose, side split, and sit-and-reach)	<60% HRmax	30 s each side	<10 s	muscle stretching (upper trapezius stretch, lunge with spinal twist, cobra pose, side split, and sit-and-reach)	<60% HRmax	30 s each side	<10 s

Table 2 demonstrates that there were no significant differences between the two groups for demographic characteristics and physical fitness variables in the baseline test (*p* > 0.05). All participants completed the prescribed training program. Significant differences between different time points across groups were evaluated using the generalized estimating equation (GEE) method. Table 3 presents the descriptive statistics of physical fitness variables for the two groups at various time points. Significant improvements across all physical fitness variables were observed in the experimental group (EG) between each time point (baseline, post-test 1, and post-test 2), while the control group (CG) showed moderate improvements with smaller effect sizes. Compared to CG, EG exhibited notably larger effect sizes post-intervention: push-up performance (d = 1.34 vs. d = 0.75), standing long jump (d = 0.90 vs. d = 0.21), jump rope (d = 1.72 vs. d = 0.98), 30 m sprint (d = 0.93 vs. d = 0.21), and sit-and-reach flexibility (d = 0.63 vs. d = 0.33).

**Table 2:** Baseline comparisons between groups

Variables	EG (*n* = 30)	CG (*n* = 30)	t-value	*p* value
	Mean (SD)	Mean (SD)		
Age (year)	18.53 (0.86)	18.60 (0.89)	−0.30	0.77
Height (cm)	175.00 (1.26)	175.10 (1.29)	−0.30	0.76
Weight (kg)	69.90 (1.16)	70.07 (1.41)	−0.50	0.62
Training background (month)	61.47 (4.49)	60.80 (4.25)	0.59	0.56
Push-ups	43.06 (7.26)	42.67 (7.48)	0.21	0.83
Standing long jump	238.00 (15.01)	238.17 (18.31)	−0.04	0.97
Jumping rope	89.77 (6.33)	90.50 (5.72)	−0.47	0.64
Sprint 30 m	4.86 (0.15)	4.87 (0.15)	−0.16	0.87
Sit-and-reach	15.32 (1.95)	15.97 (1.77)	−1.35	0.18

SD, standard deviation; EG, experimental group; CG, control group.

**Table 3:** Descriptive statistics (mean, SD) of physical fitness between groups across the time

Variable	Group	Baseline	Week 6	Week 12	*p* value	Effect size (d)
		Mean (SD)	Mean (SD)	Mean (SD)		
Push-ups	EG	43.06 (7.26)	44.60 (6.53)	51.73 (5.61)	<0.001	1.34
	CG	42.67 (7.48)	43.73 (7.22)	47.77 (5.99)	<0.001	0.75
Standing long jump	EG	238.00 (15.01)	242.53 (12.37)	249.73 (10.61)	<0.001	0.90
	CG	238.17 (18.31)	239.23 (17.77)	241.80 (16.56)	<0.001	0.21
Jumping rope	EG	89.77 (6.33)	93.00 (6.25)	100.77 (6.46)	<0.001	1.72
	CG	90.50 (5.72)	92.03 (4.66)	95.87 (5.25)	<0.001	0.98
Sprint 30 m	EG	4.86 (0.15)	4.81 (0.13)	4.72 (0.15)	<0.05	0.93
	CG	4.87 (0.15)	4.87 (0.15)	4.84 (0.14)	<0.05	0.21
Sit-and-reach	EG	15.32 (1.95)	15.70 (1.80)	16.51 (1.84)	<0.001	0.63
	CG	15.97 (1.77)	16.24 (1.71)	16.54 (1.73)	<0.001	0.33

SD, standard deviation; EG, experimental group; CG, control group.

The GEE results showed no significant difference between the two groups for push-ups (χ2 = 1.09, *p* = 0.30), standing long jump (χ^2^ = 0.91, *p* = 0.34), jumping rope (χ^2^ = 1.46, *p* = 0.23), 30 m sprint (χ2 = 2.88, *p* = 0.09), and sit-and-reach scores (χ2 = 0.80, *p* = 0.37). However, the effect of time on push-ups (χ^2^ = 356.55, *p* < 0.001), standing long jump (χ2 = 148.27, *p* < 0.001), jumping rope (χ2 = 358.38, *p* < 0.001), 30 m sprint (χ2 = 144.31, *p* < 0.001), and sit-and-reach (χ2 = 284.17, *p* < 0.001) is statistically significant. The interaction between time and groups was statistically significant in push-up (χ^2^ = 26.54, *p* < 0.001), standing long jump (χ^2^ = 40.74, *p* < 0.001), jumping rope (χ^2^ = 41.95, *p* < 0.001), 30 m sprint (χ^2^ = 41.90, *p* < 0.001), and sit-and-reach (χ^2^ = 43.87, *p* < 0.001), indicating significant differences in scores between the two groups at different times (baseline, week 6, and week 12). More information can be found in Table 4.

**Table 4:** Results of GEE on physical fitness scores

Variable	Source	Wald chi-square	df	*p* value
Push-ups	group	1.09	1	0.30
	time	356.55∗	2	<0.001
	group∗time	26.54∗	2	<0.001
Standing long jump	group	0.91	1	0.34
	time	148.27∗	2	<0.001
	group∗time	40.74∗	2	<0.001
Jumping rope	group	1.46	1	0.23
	time	358.38∗	2	<0.001
	group∗time	41.95∗	2	<0.001
Sprint 30 m	group	2.88	1	0.09
	time	144.31∗	2	<0.001
	group∗time	41.90∗	2	<0.001
Sit-and-reach	group	0.80	1	0.37
	time	284.17∗	2	<0.001
	group∗time	43.87∗	2	<0.001

df, degree of freedom; ∗*p* < 0.05, significance level.

### Effects of 12-week HIFT on physical fitness measurement

The Bonferroni post hoc test was used to assess differences in physical fitness variables (strength, power, endurance, speed, and flexibility) at three time points (baseline, post-test 1, and post-test 2) between two groups of male Wushu routine athletes. The results are shown in Table 5. At post-test 2, statistically significant differences between EG and CG were found in push-up (*p* = 0.01, confidence interval [CL] = 1.08–6.85), standing long jump (*p* = 0.03, CL = 1.02–14.85), jump rope (*p* = 0.001, CL = 1.97–7.83), and 30 m sprint (*p* = 0.001, CL = −0.19–0.05) scores, while sit-and-reach flexibility showed no significant difference (*p* = 0.95). Significant improvements were observed in both groups across all outcomes. EG demonstrated greater gains than CG in muscular endurance, horizontal jump performance, rope jumping, and sprint speed, with medium-to-large effect sizes. Flexibility improved similarly in both groups, with no between-group difference at week 12.

**Table 5:** Comparison of mean scores for physical fitness between groups at three times

Variable	Test	(I) Group	(J) Group	Mean difference (I-J)	SE	*p* value	95% CL for difference		Effect size (d)
							Lower	Upper	
Push-ups	baseline	EG	CG	0.40	1.87	0.83	−3.27	4.07	0.05
	week 6	EG	CG	0.87	1.75	0.62	−2.56	4.29	0.13
	week 12	EG	CG	3.97∗	1.47	0.01	1.08	6.85	0.68
Standing long jump	baseline	EG	CG	−0.17	4.25	0.97	−8.50	8.16	0.01
	week 6	EG	CG	3.30	3.89	0.40	−4.32	10.92	0.22
	week 12	EG	CG	7.93∗	3.53	0.03	1.02	14.85	0.57
Jumping rope	baseline	EG	CG	−0.73	1.53	0.63	−3.73	2.27	0.12
	week 6	EG	CG	0.97	1.40	0.49	−1.78	3.71	0.18
	week 12	EG	CG	4.90∗	1.49	0.001	1.97	7.83	0.83
Sprint 30 m	baseline	EG	CG	−0.01	0.04	0.86	−0.08	0.07	0.07
	week 6	EG	CG	−0.06	0.04	0.11	−0.13	0.01	0.43
	week 12	EG	CG	−0.12∗	0.04	0.001	−0.19	−0.05	0.83
Sit-and-reach	baseline	EG	CG	−0.65	0.47	0.17	−1.58	0.28	0.35
	week 6	EG	CG	−0.54	0.45	0.22	−1.42	0.33	0.31
	week 12	EG	CG	−0.03	0.45	0.95	−0.92	0.86	0.02

CL, confidence interval; EG, experimental group; CG, control group; ∗*p* < 0.05, significance level.

## Discussion

This study investigated the effects of a 12-week HIFT program on physical fitness outcomes in male Wushu routine athletes. The findings indicate that HIFT was more effective than standard training in improving muscular endurance, horizontal jump performance, rope-jumping coordination, and sprint speed, while flexibility improvements were comparable between groups. These results extend previous research on HIFT in combat sports^8^^,^^9^^,^^10^ and provide the first controlled evidence for its application in Wushu.

### Muscular endurance and horizontal jump performance

Athletes in the HIFT group showed significantly greater gains in push-up performance. This improvement is likely linked to the high training volume, repeated upper-body loading, and short rest intervals embedded in As Many Rounds As Possible-style sessions. Such training produces substantial metabolic stress and promotes peripheral fatigue tolerance, contributing to enhanced muscular endurance. Similar adaptations have been reported in athletes exposed to multimodal high-intensity training.^24^

The superior improvement in standing long jump performance further suggests that HIFT effectively stimulates lower-limb power development. Although maximal power was not directly assessed, HIFT incorporates plyometric elements and weightlifting derivatives that challenge the stretch-shortening cycle and may enhance neural drive, motor-unit recruitment, and intermuscular coordination. These mechanisms align with previous findings in Taekwondo and volleyball athletes showing increased explosive performance following functional high-intensity training.^9^^,^^23^

From a sport-specific perspective, these improvements may hold practical relevance for Wushu. Horizontal jump capacity and upper-body muscular endurance have been linked to the execution quality of aerial techniques, landing stability, and connection difficulty in competitive routines. While technical performance was not directly assessed in the present study, the physical qualities improved by HIFT are theoretically transferable to key determinants of competition scoring. Future work should incorporate biomechanics-based assessments or routine scoring to verify this transfer.

### Coordination and speed

The substantial gains in rope-jumping coordination observed in the HIFT group suggest that the multimodal, rhythm-dependent nature of the training may enhance neuromuscular synchronization, movement efficiency, and fatigue resistance. HIFT requires athletes to perform complex, full-body movements at high intensities, potentially fostering improved sensorimotor integration and coordination.^10^ These findings suggest that the multimodal, intermittent nature of HIFT may enhance both muscular endurance and movement coordination. The HIFT program’s impact on lactate tolerance and oxygen transport would help athletes manage fatigue more effectively, a critical factor in Wushu’s demanding sequences.^25^

Similarly, sprint speed improved more in the HIFT group, consistent with evidence from combat sports where HIFT has been shown to enhance acceleration and short-distance running performance.^9^ This effect is likely explained by the inclusion of explosive drills and multi-joint exercises, which may improve motor-unit recruitment and rate of force development. While these mechanisms were not directly assessed in this study, prior research indicates that high-intensity, whole-body training could enhance neuromuscular responsiveness and movement economy.^26^ It is important to note that these mechanisms are inferred rather than directly measured, as physiological indices such as electromyographic activity, lactate thresholds, or oxygen uptake were not collected. Future studies should incorporate laboratory-based neuromuscular and metabolic assessments to clarify causal pathways.

### Flexibility

Both groups demonstrated improvements in sit-and-reach scores, but no between-group difference was observed. This indicates that HIFT, despite integrating dynamic mobility elements, does not confer additional benefits to static hamstring and lumbar flexibility beyond those achieved through regular training. Moreover, the sit-and-reach test captures only sagittal-plane posterior chain flexibility and does not reflect the multidirectional joint mobility required in Wushu, such as hip abduction during kicks, thoracolumbar rotation, or dynamic overhead range of motion. This limitation underscores the need for future studies to include sport-specific flexibility assessments, such as hip joint goniometry, kicking height analysis, or multi-planar mobility testing, to more comprehensively evaluate flexibility adaptations.

This 12-week randomized trial found that HIFT enhanced muscular endurance, horizontal jump performance, rope-jumping endurance, and sprint speed in male Wushu athletes, while flexibility improvements did not differ significantly from standard training. These findings suggest that HIFT is an effective supplementary approach for developing explosive and endurance-related capacities relevant to Wushu performance. It is important to note that while physical fitness gains are statistically robust, their direct impact on competitive Wushu outcomes remains inferred rather than measured, and mechanisms underlying improvements are supported by prior literature but not direct physiological assessments. Caution is warranted given the male-only sample, potential differences in training load, reliance on field-based assessments, and limited intervention duration. Future research should include diverse populations, employ gold-standard performance measures, and assess the transfer of training gains to competitive outcomes.

### Limitations of the study

Several limitations should be acknowledged. First, the study included only male university-level athletes (sub-elite, 3–5 years of training), so findings are not generalizable to female athletes or elite performers (with higher baseline fitness levels that may reduce adaptation magnitude). Second, although intensity was monitored using heart-rate sensors, total training volume (e.g., Rate of Perceived Exertion, tonnage, and repetitions) was not fully quantified. Thus, differences in overall workload between groups may partially explain the observed effects. Third, outcomes were assessed with field-based tests rather than laboratory gold standards (e.g., isokinetic dynamometry and force plates), reducing physiological inference precision. Fourth, flexibility was assessed only with the sit-and-reach test, which does not capture Wushu’s dynamic flexibility demands. Finally, the 12-week intervention period may not capture the long-term sustainability of HIFT-induced adaptations.

Future research should extend intervention duration to 24–36 weeks and incorporate 3–6 months follow-up assessments to evaluate the sustainability of HIFT-induced adaptations. Studies should also include female and elite athletes to enhance generalizability and examine sex- or level-specific responses. Moreover, integrating laboratory-based measures such as electromyography (EMG), lactate threshold, VO_2_max testing, and force-plate kinematics would allow verification of proposed neuromuscular and metabolic mechanisms. Sport-specific performance outcomes, including video-based technical scoring of jump height, rotational completion, and landing quality, as well as competition results, should be incorporated to directly link fitness gains with Wushu performance. Finally, future studies should systematically capture objective workload metrics to better isolate the specific effects of HIFT from general training load differences.

## Resource availability

### Lead contact

Further information and requests for resources and materials should be directed to and will be fulfilled by the lead contact, Qian Lei (catel7508@gmail.com).

### Materials availability

This study did not generate new unique reagents or materials.

### Data and code availability

• All data reported in this paper will be shared by the lead contact upon reasonable request.
• No custom code or software was generated or used in this study.
• Any additional information required to reanalyze the data reported in this paper is available from the lead contact upon request.

## Acknowledgments

We extend our sincere gratitude to the participants and coaches for their enthusiastic involvement.

This study was funded by the Sichuan Research Center for Innovation and Development of Traditional Ethnic Minority Sports with the code AS-XJPT2024-05.

## Author contributions

X.Z. and S.M. contributed to conceptualization, investigation, visualization, writing – original draft, and writing – review and editing. K.G.S. was involved in investigation, supervision, and writing review. F.X., D.Z., and Q.L. supported resources and supervision. All authors have read and approved the final version of the manuscript and have agreed on the order of authorship for submission.

## Declaration of interests

The authors declare no competing interests.

## STAR★Methods

### Key resources table

**Table:** 

REAGENT or RESOURCE	SOURCE	IDENTIFIER
**Human participants**		

Male Wushu routine athletes	This study	See STAR Methods: Subject Details

**Deposited data**		

Physical fitness datasets generated in this study	https://data.mendeley.com/drafts/5yfrpr437h	Deposited in Mendeley Data (DOI available upon publication)

**Software and algorithms**		

SPSS Statistics version 27.0	IBM Corporation	https://www.ibm.com/products/spss-statistics
G∗Power version 3.1	Heinrich Heine University Düsseldorf	https://www.psychologie.hhu.de/arbeitsgruppen/allgemeine-psychologie-und-arbeitspsychologie/gpower
Microsoft Excel	Microsoft Corporation	N/A

**Other**		

Polar H10 heart rate sensor	Polar Electro Oy, Finland	https://www.polar.com
Standard Wushu training equipment (e.g., mats, ropes)	Local suppliers	N/A
High-intensity functional training (HIFT) protocol	This study	Described in STAR Methods: Intervention Procedures

### Experimental model and study participant details

This study was conducted as a randomised controlled trial with repeated measures at baseline, week 6, and week 12. Two universities in Hebei Province, China, were invited to participate. Because only one cluster per arm was available, the design does not qualify as a cluster-randomised controlled trial; instead, participants were individually randomised within each site to ensure balanced allocation. Randomisation was performed by an independent researcher using a computer-generated sequence with allocation concealment via sealed opaque envelopes. Outcome assessors and video scorers were blinded to group assignment.

Seventy healthy male college Wushu routine athletes were recruited. Inclusion criteria were: (1) ≥3 years of continuous Wushu training; (2) no musculoskeletal injuries in the past 6 months; (3) consistent training attendance (>85% of weekly sessions); and (4) willingness to refrain from additional structured resistance or endurance training during the study. Exclusion criteria were: (1) current injury or chronic illness, (2) use of performance-enhancing drugs or supplements, and (3) failure to meet baseline testing requirements. Ten athletes were screened but excluded for failing to meet the criteria. Written informed consent was obtained from all participants. Therefore, only 60 eligible participants were randomly divided into EG (*n* = 30, age = 18.53 ± 0.86 years, weight = 69.90 ± 1.16 kg, height = 175.00 ± 1.26 cm, training experience = 61.47 ± 4.49 months) and CG (*n* = 30, age = 18.60 ± 0.89 years, weight = 70.07 ± 1.41 kg, height = 175.10 ± 1.30 cm, training experience = 60.80 ± 4.25 months). The Wushu routine training course includes physical fitness training and technical training. During the experimental intervention period, all participants participated in the training throughout the entire process.

### Method details

We followed the “Consolidated Standards of Reporting Trials” guidelines (Figure 1) when drafting this manuscript. In addition, the experimental protocol for this study has been registered with the Clinical Trials Government Protocol and the Results System Receipt (Clinical Trials. Gov; ID: NCT06181487).

#### Sample size calculation

Sample size was estimated using G∗Power 3.1 based on a repeated-measures ANOVA design (f = 0.25, α = 0.05, power = 0.80, correlation = 0.5, non-sphericity = 1). This yielded a minimum of 52 participants; 60 were recruited to account for potential dropouts. Although the final analysis used Generalised Estimating Equations (GEE), a Monte Carlo simulation confirmed that 60 participants provided ≥0.80 power to detect moderate group × time interactions under the assumed correlation structure.

#### Intervention details

##### Experimental group (HIFT)

The experimental group (EG) completed a 12-week HIFT program with 3 sessions per week, 60 min per session. Each session included:

Warm-up (10 min): dynamic mobility, light jogging, activation drills.

Main session (40 min): multi-station circuit performed in AMRAP style (as many rounds as possible) with progressive overload. Week circuit: Clean & Jerk (40–50% 1RM, 8 reps), Box Jumps (10 reps), Push-ups (15 reps), Kettlebell Swings (12 reps, 16 kg), Rope Jumping (40 s), Plank Hold (30 s). Work: rest ratio started at 40:20s and progressed to 50:10s by week 12. Training intensity was monitored using Polar H10 heart-rate sensors, targeting 80–90% HRmax. Load progression: weights increased by 5–10% every 2–3 weeks or when athletes completed >90% of assigned reps.

Cool-down (10 min): stretching and relaxation.

Adherence was tracked by attendance logs (>90% compliance achieved).

#### Control group (standard training)

The control group (CG) continued standard physical conditioning matched for frequency and duration (3 × 60 min/week). Sessions included: jogging, callisthenics (push-ups, sit-ups), bodyweight squats, rope skipping, and static stretching. Intensity was monitored by HR sensors, targeting 60–70% HRmax. Unlike HIFT, the program followed a fixed repetition scheme (8–12 reps, 3 sets, 60 s rest) without progressive overload.

#### Outcome measures

All tests were conducted in an indoor sports hall at baseline, week 6, and week 12 under standardised conditions (temperature 22°C–24°C, same surface, same footwear). Athletes performed a 10-min standardised warm-up before testing. For each test, two familiarisation trials were given; the best of two valid attempts was recorded. Assessors were blinded to group assignment.

##### Muscular endurance (push-up test)

Maximum number of push-ups in 60 s at a metronome pace (40 bpm). Hands shoulder-width, chest to 5 cm above floor, full arm extension required. Reliability: ICC = 0.91.

##### Horizontal jump performance (standing long jump)

Distance measured in cm from the take-off line to the heel of the landing foot. Two attempts, best recorded. ICC = 0.89.

##### Coordination (1-min rope jumping)

Number of consecutive jumps completed in 60 s with a standardised rope length. Errors discounted. ICC = 0.87.

##### Speed (30 m sprint)

Measured with dual-beam photocell timing gates (Brower Timing Systems, Utah, USA). Best of two trials recorded. ICC = 0.94.

##### Flexibility (sit-and-reach)

Assessed with a standard sit-and-reach box. Three attempts, best recorded. ICC = 0.90.

### Quantification and statistical analysis

All data were analyzed with SPSS v27.0. Descriptive statistics are reported as mean ± SD. Group differences across time were analyzed using Generalised Estimating Equations (GEE) with robust standard errors, an identity link, and exchangeable correlation structure. GEE was selected because it is robust for repeated-measures data with potential non-sphericity and accounts for within-subject correlations without requiring strict assumptions about data distribution. Compared with repeated-measures ANOVA, GEE provides stable parameter estimates even when covariance structures are violated, making it suitable for longitudinal sports-science data collected at multiple time points. Additionally, GEE allows modeling of interaction effects (group × time) using all available cases while providing robust standard errors. Primary effects of interest were time × group interactions. Model-based effect sizes (Cohen’s d) were calculated from estimated marginal means, standardised by baseline SD, and 95% confidence intervals (CIs) for effect sizes are provided in Table S1. Missing data were not present. Statistical significance was set at *p* < 0.05.

### Additional resources

Before data collection, this study has been reviewed and approved by the Ethics Committee of Universiti Putra Malaysia (JKEUPM-2023-1359).
