# Supplementary material for: Effects of 12-week high-intensity functional training on physical fitness in Wushu athletes
Source: iScience. 2026 Feb 10;29(3):114985. doi: 10.1016/j.isci.2026.114985 (PMC12961278; doi:10.1016/j.isci.2026.114985)
Supplement: Document S1. Table S1 [file mmc1.pdf]

iScience, Volume *29*

Supplemental information

Effects of 12-week high-intensity functional training on physical fitness in Wushu athletes

Xinzhi Wang, Kim Geok Soh, Shuzhen Ma, Fan Xu, Dong Zhang, and Qian Lei

Table S1: Within-Groups Comparison of Mean Scores for Physical Fitness Across the Time

Variable	Group	(I) Test	(J) Test	Mean Difference (I-J)	SE	p-value	95% CL for Difference		Effect size (d)
							Lower	Upper	
Push-ups	EG	Pretest	Posttest 1	-1.533*	0.305	<0.001	-2.130	-0.937	1.34
		Pretest	Posttest 2	-8.666*	0.617	<0.001	-9.876	-7.457	
		Posttest 1	Posttest 2	-7.133*	0.439	<0.001	-7.994	-6.273	
	CG	Pretest	Posttest 1	-1.067*	0.283	<0.001	-1.621	-0.513	0.75
		Pretest	Posttest 2	-5.100*	0.472	<0.001	-6.026	-4.174	
		Posttest 1	Posttest 2	-4.033*	0.415	<0.001	-4.847	-3.220	
Standing long jump	EG	Pretest	Posttest 1	-4.533*	0.654	<0.001	-5.816	-3.251	0.90
		Pretest	Posttest 2	-11.733*	1.172	<0.001	-14.030	-9.437	
		Posttest 1	Posttest 2	-7.200*	0.813	<0.001	-8.793	-5.607	
	CG	Pretest	Posttest 1	-1.067*	0.290	<0.001	-1.636	-0.498	0.21
		Pretest	Posttest 2	-3.633*	0.491	<0.001	-4.596	-2.671	
		Posttest 1	Posttest 2	-2.567*	0.379	<0.001	-3.310	-1.824	
Jumping rope	EG	Pretest	Posttest 1	-3.233*	0.405	<0.001	-4.0264	-2.4402	1.72
		Pretest	Posttest 2	-11.000*	0.772	<0.001	-12.512	-9.4875	
		Posttest 1	Posttest 2	-7.767*	0.526	<0.001	-8.7983	-6.7350	
	CG	Pretest	Posttest 1	-1.533*	0.373	<0.001	-2.2651	-0.8015	0.98
		Pretest	Posttest 2	-5.367*	0.460	<0.001	-6.2695	-4.4638	
		Posttest 1	Posttest 2	-3.833*	0.397	<0.001	-4.6123	-3.0544	
Sprint 30 m	EG	Pretest	Posttest 1	.047*	0.010	<0.001	0.027	0.067	0.93
		Pretest	Posttest 2	.137*	0.013	<0.001	0.111	0.162	
		Posttest 1	Posttest 2	.090*	0.005	<0.001	0.079	0.101	
	CG	Pretest	Posttest 1	-0.003	0.009	0.705	-0.021	0.014	0.21
		Pretest	Posttest 2	.027*	0.013	0.045	0.001	0.053	
		Posttest 1	Posttest 2	.030*	0.008	<0.001	0.014	0.046	
Sit-and-reach	EG	Pretest	Posttest 1	-.373*	0.055	<0.001	-0.482	-0.265	0.63
		Pretest	Posttest 2	-1.190*	0.095	<0.001	-1.377	-1.003	
		Posttest 1	Posttest 2	-.817*	0.075	<0.001	-0.963	-0.670	
	CG	Pretest	Posttest 1	-.267*	0.043	<0.001	-0.351	-0.183	0.33
		Pretest	Posttest 2	-.570*	0.045	<0.001	-0.658	-0.482	
		Posttest 1	Posttest 2	-.303*	0.028	<0.001	-0.359	-0.248	

Noted: EG: experimental group; CG: control group; *p<0.05 level of significance.
